# 早期非小细胞肺癌：手术与放疗孰优孰劣？

**DOI:** 10.3779/j.issn.1009-3419.2015.06.14

**Published:** 2015-06-20

**Authors:** 慧 刘

**Affiliations:** 510060 广州，中山大学肿瘤防治中心放疗科 Department of Radiotherapy, Sun Yat-sen University Cancer Center, Guangzhou 510060, China

最近，美国MD Anderson癌症中心放疗科张玉蛟教授在*The Lancet Oncology*杂志发表了一项关于可手术Ⅰ期非小细胞肺癌的临床研究^[1]^结果。研究结果来源于因病例入组缓慢而提前关闭的两项Ⅲ期前瞻性随机临床试验（STARS：NCT00840749和ROSEL：NCT00687986）。入组标准为临床分期T1-2a（< 4 cm）N0M0，评估可耐受手术的患者。观察终点为总生存期。该研究最终有58例患者进入分析，其中31例患者接受立体定向放射治疗（stereotactic ablative radiotherapy, SABR），27例患者接受根治性手术。患者在入组前均接受严格的治疗前评估。

随访结束后，SABR组患者3年预期总生存率为95%，手术组为79%（*P*=0.037），SABR组有1例而手术组有6例患者死于随访周期内。SABR组患者3年无复发生存率为86%，手术组为80%（*P*=0.54）。SABR组有1例原发灶复发，4例区域淋巴结复发，1例远处转移；手术组有1例区域淋巴结复发及2例远处转移。治疗并发症方面，SABR组有3例出现3度治疗毒性（3例胸痛，2例呼吸困难或咳嗽，1例疲劳及肋骨骨折），手术组中1例患者术后并发症死亡，12例患者出现3度-4度治疗毒性（其中4例呼吸困难，4例胸痛，2例肺部感染）。

研究结果提示，SABR对于可手术的Ⅰ期非小细胞肺癌是一种安全有效的治疗方式，可做为手术治疗之外的另一种局部治疗选择。该结果发布后，引起多方讨论，这项研究有以下几个方面值得思考：

① 来源于两个提前关闭的临床试验、样本量小、随访周期短，为什么*The Lancet Oncology*同意发表？实验设计简单明了，目的在于直接比较目前两种主流局部治疗模式对Ⅰ期非小细胞肺癌的疗效、失败模式及毒性。其结果将直接影响到患者治疗模式的改变，对临床医生决策、患者选择以及医保体系均有长远影响。

② 两组患者3年生存期有统计学差异，是否可以就此对SABR及手术的治疗结果下结论？由于该研究样本量小，随访时间短，此处的P值差异不足以得到绝对的结论。同时，根据既往的研究结果，Ⅰ期非小细胞肺癌经过根治性手术后，最初4年的复发危险为6%-10%每人每年，复发几率约为5%-10%，远处转移率约为10%-20%。即使在该研究中，手术组的复发患者的绝对数仍低于SABR组。因此，手术由于对原发灶及区域淋巴结进行了切除，其局部及区域复发率较SABR低，这是公认的情况。此外，区域淋巴结清扫的结果，尚可以对患者下一步治疗提供参考依据，这也是目前其他方法不能取代的。

然而，手术作为一种开放性、有创性的局部治疗方式，其风险相对无创的SABR必然较高，对患者的一般情况及手术医师的操作技巧有较高的要求。根据荟萃分析的结果，患者接受微创手术术后30天-90天3度以上并发症发生率约为2%-5.4%，而SABR仅为0.7%。该研究的结果也与既往数据相符，SABR有较少的重度并发症发生率。该组患者的生存率能高于手术组，主要来源于较少的术后及围术期死亡率。

③ 尽可能提高治疗前评估的准确性。对肺癌患者开始治疗之前，详细的评估是最重要的，这也是目前常常不能得到重视的一个关键步骤。不合适的评估将导致患者进入不恰当的治疗。该研究所有患者都接受正电子发射型计算机断层显像（positron emission computed tomography, PET）-计算机断层扫描（computed tomography, CT）检查，可疑区域淋巴结均接受支气管内超声（endobronchial ultrasound, EBUS）或纵隔镜的进一步确认。临床治疗前的准确评估对于非手术局部治疗至关重要。除了临床分期之外，SABR前的评估还包括患者一般情况、心肺功能以及肿瘤部位。张玉蛟教授的研究特别提到中央型病灶与周围型病灶的剂量差别，中央型靠近肺门、纵隔，STARS试验中总剂量50 Gy/4次，每次12.5 Gy，而ROSEL则为60 Gy/5次，每次12 Gy。而外周型病灶因远离肺门及纵隔，安全性较高，总剂量在两个试验中均为54 Gy/3次，每次18 Gy。

④ SABR的执行需要充分的物理技术保障。近20年来放疗的进步得益于物理技术的充分发展，目前我院的SABR每日执行误差平均可控制在2 mm-3 mm以内。SABR对于早期非小细胞肺癌的优势在于无创、低毒，若不能在物理上做好充分的质量保证和质量控制，单次放疗剂量超过10 Gy是非常危险的。

综上所述，对于一般状况良好的Ⅰ期非小细胞肺癌患者而言，目前手术仍然是标准治疗手段。近年由于物理技术的进步，SABR已经能达到与手术相近的局部控制效果，在SABR治疗局部失败的患者中，可以采用挽救性手术继续处理。手术和SABR作为两种主流的局部治疗方法，由于技术上的差异，各有其优缺点，尚需大宗随机对照临床试验数据进一步证实。但与其纠结于孰优孰劣，个人认为，结合患者的实际情况，选择最合适的局部治疗方法，也许才是未来的方向。
